# Supplementary material for: ETV7 regulates breast cancer stem-like cell features by repressing IFN-response genes
Source: Cell Death Dis. 2021 Jul 27;12(8):742. doi: 10.1038/s41419-021-04005-y (PMC8316333; doi:10.1038/s41419-021-04005-y)
Supplement: Supplementary file 1 — Suppl Fig and Table Legends [file 41419_2021_4005_MOESM1_ESM.docx]

**Suppl. Fig. 1.** A) Western blot analysis of the different expression of ETV7 among various breast cancer-derived cell lines. On the right of each blot is indicated the approximate observed molecular weight. B) Western blot analysis of ETV7 expression in T47D cells transfected with siRNA #1 and siRNA # 2 against ETV7 or the relative scrambled control for 72 h. HSP70 was used as a loading control. On the right of each blot is indicated the approximate observed molecular weight. C) Cell Titer Glo assay for survival analysis upon the treatment with 5-FU (0.25 and 0.5mM) in T47D cells transfected with siETV7 #1 and siETV7 #2 or the scrambled control. D) A representative dotplot of the flow cytometry analysis performed on MCF7 Empty and MCF7 ETV7 cells treated with 5-FU 200 μM for 72 hours on the right and the relative percentage of Annexin V positive cells calculated as the difference of 5-FU and DMSO treated cells on the left. E) RT-qPCR analysis of ABCB1, ABCC1, and ABCG2 expression in T47D Empty and T47D ETV7 cells. Bars represent the averages and standard deviations of two independent experiments. F) Relative percentage of Annexin V positive cells calculated as a difference of 5-FU and DMSO treated cells measured by Annexin V-FITC/PI staining of T47D Empty and T47D ETV7 cells treated with 5-FU 1 mM for 72 hours. Bars represent the averages and standard deviations of two independent experiments. G) RT-qPCR analysis of ABCB1, ABCC1, and ABCG2 expression in T47D Empty and T47D ETV7 cells. H-I) Cell Titer Glo assays for survival analysis upon treatment with 5-FU alone or in combination with ABC transporter inhibitor (Elacridar) (H) or with BCL-2 inhibitor (Navitoclax) (I) in MCF7 ETV7 cells. J) Western Blot analysis of the anti-apoptotic BCL-2 and Survivin protein levels in T47D Empty and T47D ETV7 cells. Tubulin was used as loading control. On the right of each blot is indicated the approximate observed molecular weight. Bars represent the averages and standard deviations of at least three independent experiments. * = p-value < 0.05; ** = p-value < 0.01; *** = p-value < 0.001.

**Suppl. Fig. 2.** A-B) ViCell Assay for survival analysis upon radiotherapy treatment in MCF7 (A) and T47D (B) cells over-expressing ETV7 and their empty control. C-D) Annexin V-FITC/PI analysis of MCF7 (C) and T47D (D) cells Empty or over-expressing ETV7 treated with radiotherapy with 2-6-10 Gy for 72 hours. The relative percentage of Annexin V positive cells was calculated as the difference between treated and untreated cells. Bars represent the averages and standard deviations of at least four independent experiments. E-F) Doubling time of MCF7 (E) and T47D (F) Empty and ETV7 cells calculated by cell count at ViCell instrument. Bars represent the averages and standard deviations of at least four independent experiments. * = p-value < 0.05; ** = p-value < 0.01.

**Suppl. Fig. 3.** A-B) RT-qPCR analysis of CD44 and CD24 expression in MCF7 Empty and MCF7 ETV7 (A) and in T47D Empty and T47D ETV7 (B) cells. Bars represent the averages and standard deviations of at least three independent experiments. C-D) ALDEFLUOR analysis in MCF7 (C) and T47D (D) Empty and ETV7 cells. The histogram on the left summarizes the percentage of ALDH positive cells in Empty and ETV7 over-expressing cells; on the right, a representative dot plot of the results obtained at FACS Canto II. E) Western blot analysis of EpCAM expression in T47D Empty and ETV7 cells. HSP70 was used as a loading control. On the right of each blot is indicated the approximate observed molecular weight. F) RT-qPCR analysis of ETV7 expression in MDA-MB-231 transfected with siRNA against ETV7 (siETV7 #1 and #2) and the relative scramble control for 72 hours. Bars represent the averages and standard deviations of at least three independent experiments. * = p-value < 0.05; *** = p-value < 0.001 G) Western blot analysis of ETV7 expression in MDA-MB-231 cells transfected with siRNA #1 and siRNA # 2 against ETV7 and the relative scramble control for 72 h. HSP70 was used as a loading control. On the right of each blot is indicated the approximate observed molecular weight. H) Representative dot plot of CD44-APC and CD24-FITC staining and flow cytometry analysis in MDA-MB-231 cells transfected with siRNA against ETV7 (siETV7 #1 and #2) and the relative scramble control for 72 hours. I) A representative image of a Tracking by Indel DEcomposition (TIDE) online tool analysis output of the selected SK-BR-3 clone following ETV7 gene editing using CRISPR-Cas9 technology and clonal selection. J) CD44-APC and CD24-FITC staining and flow cytometry analysis in SK-BR-3 parental and ETV7 knock-out cells. On the left the histogram quantifying the percentage of CD44^+^/CD24^-^ population in the parental and ETV7 knock-out cells; on the right, a representative dotplot of the results obtained at FACS Canto A. Bars represent the averages and standard deviations of two biological replicates.

**Suppl. Fig. 4.** A) Gene ontology analysis of commonly down-regulated DEGs in MCF7 and T47D cells (ETV7 vs. Empty). The number and fraction of commonly down-regulated DEGs that are annotated with a specific GO category are indicated by the dot size and on the x-axis, respectively. Dots are color-coded based on the enrichment adjusted p-values. In the image are shown the top 10 significant terms. B) RT-qPCR for validation of genes of the ETV7-regulated IFN-responsive signature with a Fold Change (FC) < -2 in T47D Empty and ETV7 cells. C) Western blot analysis of ETV7 expression in MCF-7 cells transfected with siRNA #1 and siRNA # 2 against ETV7 and the relative scrambled control for 72 h. HSP70 was used as a loading control. On the right of each blot is indicated the approximate observed molecular weight. D-F) RT-qPCR analysis of the expression of IFN-responsive genes in T47D (D), MDA-MB-231 (E), and SK-BR-3 (F) cells transfected with ETV7 targeting siRNA #1 and siRNA #2 or the scrambled control. Bars represent the averages and standard deviations of at least three biological replicates. G) Western blot analysis of ETV7 expression in SK-BR-3 cells transfected with siRNA #1 and siRNA # 2 against ETV7 and the relative scramble control for 72 h. HSP70 was used as a loading control. On the right of each blot is indicated the approximate observed molecular weight. H) RT-qPCR analysis of the expression of IFN-responsive genes in HCC-70 cells transfected with ETV7 targeting siRNA #1 and siRNA #2 or the scramble control. Bars represent the averages and standard deviations of at least three biological replicates. I) Western blot analysis of the ETV7 expression in HCC-70 cells transfected with siRNA #1 and siRNA # 2 against ETV7 and the relative scrambled control for 72 h. HSP70 was used as a loading control. On the right of each blot is indicated the approximate observed molecular weight. J) RT-qPCR analysis of the normalized expression of the genes regulated by ETV7 (IFITM2, CASP4, CFB, ICAM1, PARP14, PROCR) in MCF7 cells treated with 5 ng/ml IFN-β (red) or IFN-γ (blue) at different time points. Bars represent the averages and SEM of at least three biological replicates. * = p-value <0.05; ** = p-value < 0.01; *** = p-value < 0.001.

**Suppl. Fig. 5.** A-B) Percentage of third (A) and fourth (B) generation mammosphere formation efficiency (% MFE) in MCF7 Empty and ETV7 cells in response to 5 ng/ml IFN-β or IFN-γ calculated as number of mammospheres per well/number of cells seeded per well X 100. C) RT-qPCR analysis of the normalized expression of the genes regulated by ETV7 in MCF7 cells over-expressing ETV7 treated with 5ng/ml IFN-β (red) or IFN-γ (blue) at different time points. Relative Fold Change has been obtained normalizing the expression of the genes of interest relative to their expression in MCF7 Empty cells untreated. Bars represent the averages and standard deviation of at least three biological replicates. ** = p-value < 0.01.

**Suppl. Fig. 6.** A-B) Western Blot analysis of phosphorylated and total levels of STAT1 in MCF7 Empty and ETV7 cells in response to 5ng/ml IFN-β (A) or IFN-γ (B) at different time points. Tubulin expression was used as loading control. Blots were cropped for clarity and conciseness of the presentation. On the left of each blot is indicated the approximate observed molecular weight.

**Supplementary Table 1:** Sequences of primers used for RT-qPCR.

**Supplementary Table 2:** Results of GSEA analysis of MCF7 and T47D cells over-expressing ETV7 vs MCF7 and T47D controls cells for Hallmark gene sets collection. The absolute value of the Normalized Enrichment Score (NES) represents the degree of the enrichment of the gene set in one of the two groups under comparison; NES is positive if the gene set is enriched in MCF7 and T47D cells over-expressing ETV7 compared to controls, negative vice versa. Gene sets are significantly enriched with a false discovery rate (FDR) below 0.05.
