# Supplementary material for: ETV7 regulates breast cancer stem-like cell features by repressing IFN-response genes
Source: Cell Death Dis. 2021 Jul 27;12(8):742. doi: 10.1038/s41419-021-04005-y (PMC8316333; doi:10.1038/s41419-021-04005-y)
Supplement: Supplementary file 2 — Supplementary Table 1 [file 41419_2021_4005_MOESM2_ESM.pdf]

Supplementary Table 1: Sequences of primers used for RT-qPCR.

**Gene symbol**	**Forward primer**	**Reverse primer**
ETV7	CAAGATCTTCCGAGTTGTGGA	ACGACCAACACTGGGATGAT
IFITM2	CGCGTACTCCGTGAAGTCTA	GTTCACCCGGTTCTTGTGAT
IFI35	TGAGAGAGACCACAGCCCTT	GGAGGGCGGCATCCAGT
HERC6	GGAGCTGCCAGAACCAATTC	AAGACCCTTCCTTTGTGGCA
PROCR	CTCGGTATGAACTGCGGGAA	TTGTTTGGCTCCCTTTCGTG
APOL6	TTTCTCCAGCCCAGACACTC	TCAAATGATTTTCTTCTCTCCACGG
CASP4	CTGTTCCCTATGGCAGAAGGC	TCTGCCATGACCCGAACTTT
CFB	GACACGAGAGCTGTATGGGG	CTTCTCCCCTCCTACGCTGA
PARP14	TGCCAAGAATGGCCAGACAA	TATGCCACAGCATTCTTTCCG
ICAM1	ATGGCAACGACTCCTTCTCG	GCCGGAAAGCTGTAGATGGT
ABCB1	TGCCTATGGAGACAACAGCC	TGAAGGCATGTATGTTGGCCT
ABCC1	CCCGCTCTGGGACTGGAA	GTAGAAGGGGAAACAGGCCC
ABCG2	TCAGCTGGTTATCACTGTGAGG	GGCTCTATGATCTCTGTGGCT
CD44	AGCACAATCCAGGCAACTCC	CTGGTATGAGCTGAGGCTGC
CD24	GCTCCTACCCACGCAGATTTAT	GCCTTGGTGGTGGCATTAGT
YWHAZ, reference gene	CAACACATCCTATCAGACTGGG	AATGTATCAAGTTCAGCAATGGC
GAPDH, reference gene	TCCAAAATCAAGTGGGGCGA	AGTAGAGGCAGGGATGATGT
